# A Clinical Picture of Amyopathic Dermatomyositis

**DOI:** 10.7759/cureus.81246

**Published:** 2025-03-26

**Authors:** Brandon Dempsey, Dyllan Epstein, Carmel Joseph, Sahar S Amini, Allan W Bloom, Sonia Daryanani, Marc M Kesselman

**Affiliations:** 1 Medical School, Nova Southeastern University Dr. Kiran C. Patel College of Osteopathic Medicine, Davie, USA; 2 Internal Medicine, Nova Southeastern University Dr. Kiran C. Patel College of Osteopathic Medicine, Davie, USA; 3 Rheumatology, Nova Southeastern University Dr. Kiran C. Patel College of Osteopathic Medicine, Davie, USA

**Keywords:** academic rheumatology, amyopathic dermatomyositis, clinical rheumatology, dermatomyositis, inflammatory myositis

## Abstract

Clinically amyopathic dermatomyositis (CADM) is a rare subset of classic dermatomyositis (DM) with distinct clinical features and unique autoantibody profiles. Affected individuals usually present with the cutaneous signs of DM, without muscle pain, proximal weakness, or abnormal muscle labs and imaging results. Here, we present the case of a 69-year-old Caucasian male for evaluation of a positive antinuclear antibody (ANA) test and an eight-month history of a rash. He had no associated muscle weakness or pain. A physical examination noted a rash characteristic of dermatomyositis, including pathognomonic Gottron's papules. Skin biopsies were inconclusive, and labs were negative for anti-Jo1 antibodies or elevated muscle enzymes. Treatment was first initiated with hydroxychloroquine before ultimately being switched to methotrexate, which was more effective for him.

## Introduction

Clinically amyopathic dermatomyositis (CADM), also known as cutaneous DM, is a subset of idiopathic inflammatory myopathies (IIM) [1]. CADM shares many cutaneous findings with DM but lacks the characteristic findings of muscle disease for six months or longer [2]. CADM can be divided into hypomyopathic dermatomyositis (HDM) and amyopathic dermatomyositis (ADM). HDM is when there is no clinical weakness but subclinical diagnostic findings, such as laboratory, electromyography (EMG), biopsy, and imaging signs of myositis. ADM presents with no weakness or abnormal findings on myositis workup [3].

The disease is more prevalent in women, with an average age of diagnosis between 42 and 69 years [4]. Antibodies related to CADM include anti-Mi2, which is associated with the typical heliotrope rash, Gottron’s papules, V-sign, shawl sign, cuticular overgrowth, and photosensitivity [5]. Anti-CADM 140/MDA5, a more specific antibody for CADM, is associated with an increased risk of developing rapidly progressive interstitial lung disease (ILD) [3,5]. Other antibodies, including anti-aminoacyl-tRNA synthase antibodies, anti-transcription intermediary factor 1γ (TIF1-γ), anti-nuclear matrix protein 2 (NXP2), and anti-small ubiquitin-like modifier activating enzyme (SAE), are all associated with different presenting symptoms of CADM [6]. The clinical manifestation of the other autoantibodies can be widely variable, and some symptoms may present as isolated findings, such as mechanic’s hands [6].

## Case presentation

**Figure 1:**
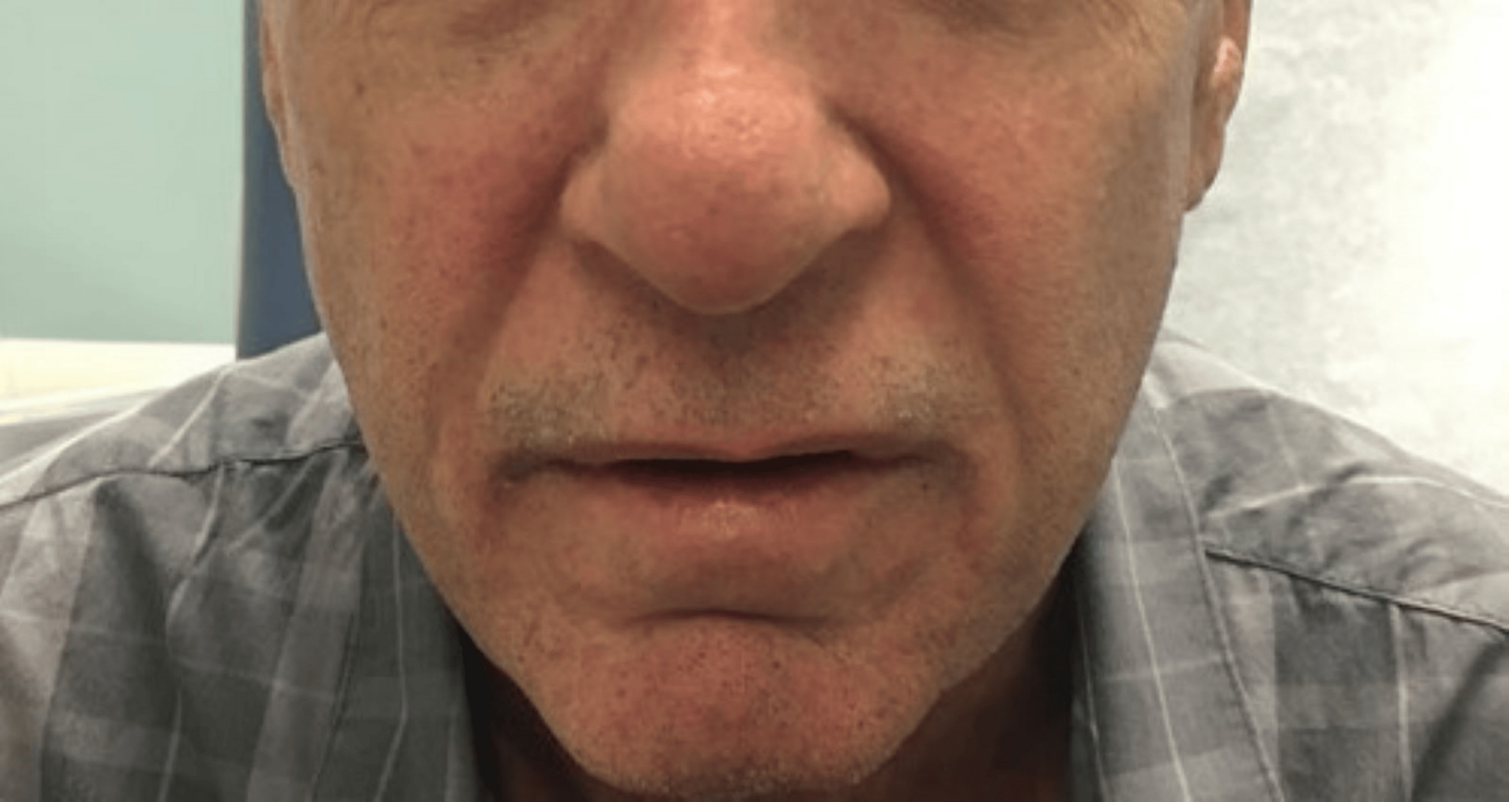
Malar rash involving nasolabial folds

**Figure 2:**
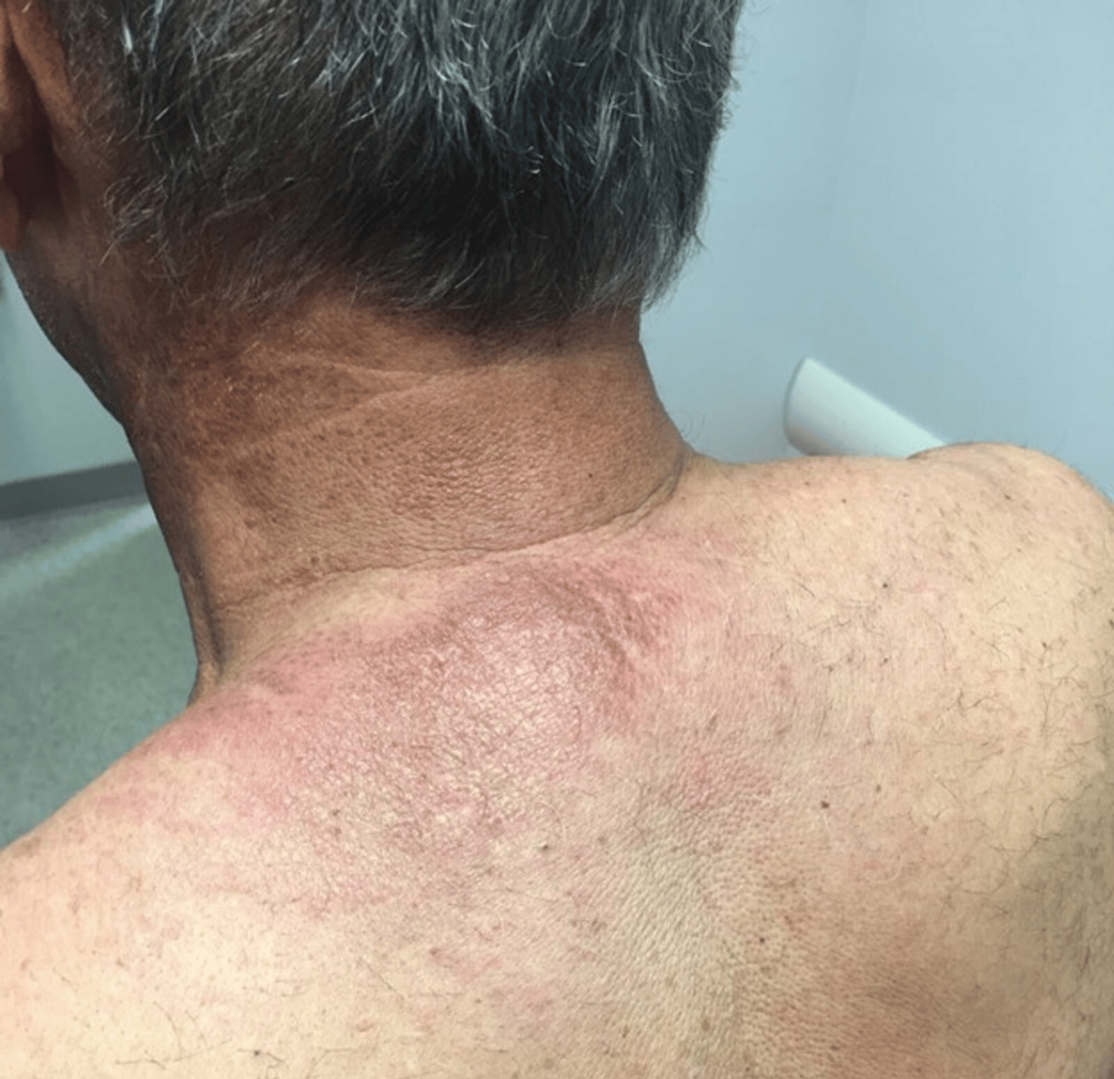
Shawl sign

**Figure 3:**
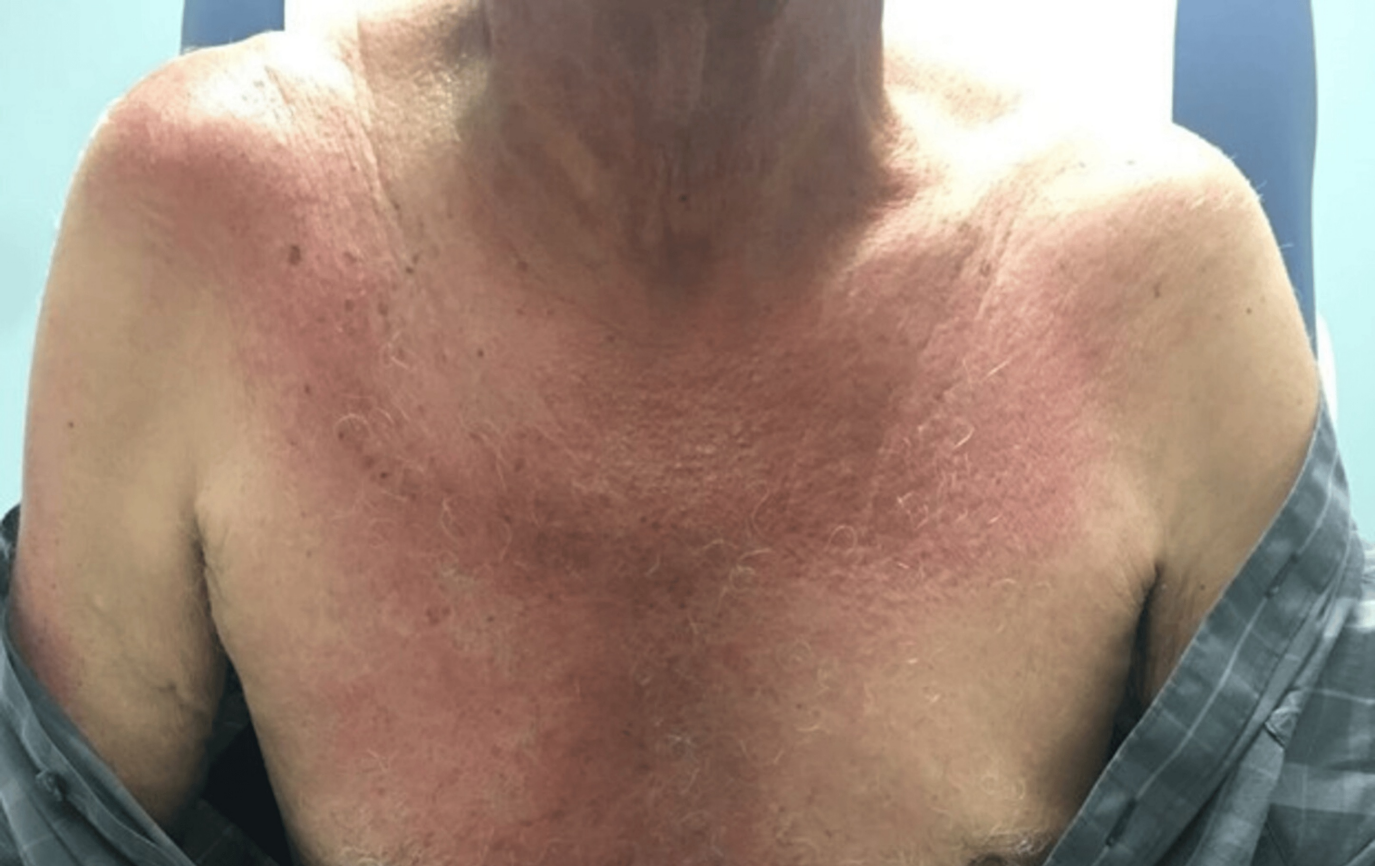
V-sign

**Figure 4:**
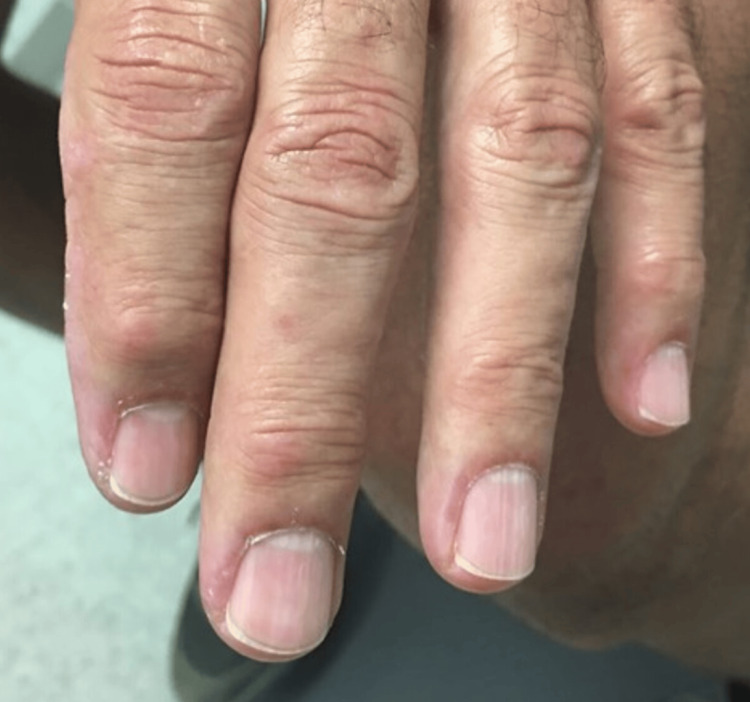
Gottron's papules and cuticular hypervascularity

**Figure 5:**
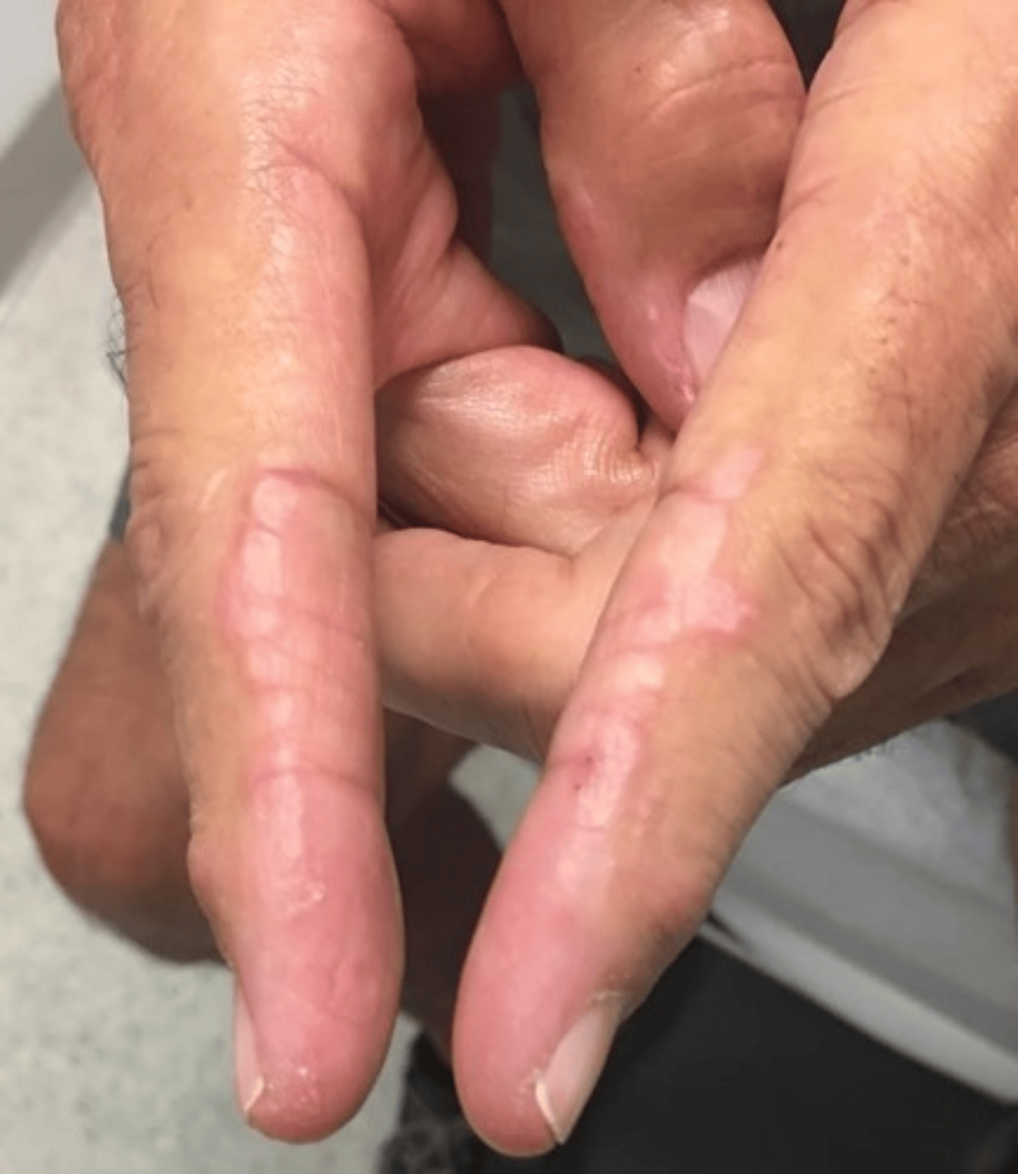
Mechanic fingers

The patient was a 69-year-old Caucasian male who presented for a rheumatological evaluation following a referral from his primary care physician due to a positive antinuclear antibody (ANA). The patient reported a rash on his chest that began eight months prior to his visit, followed by facial redness, with the rash spreading to his neck, upper back, scalp, and arms. He previously had an evaluation by a dermatologist, who performed skin biopsies that revealed findings consistent with nonspecific dermatitis without signs of immunofluorescence. Of note, a muscle biopsy was not performed at this time. He had an evaluation by an allergist, who performed a skin allergy panel for common allergens, which did not reveal any significant findings. He was prescribed a methylprednisolone dose pack that provided no relief. The patient’s past medical history was notable for longstanding benign prostatic hyperplasia, and his only current medication was tamsulosin. A review of the systems was positive for rash and skin lesions. His vital signs were all within normal limits. The physical examination revealed a pink, erythematous rash bilaterally on the face (Figure 1), as well as a pink, erythematous macular rash on the chest, neck, and upper back bilaterally (Figure 2; Figure 3). A nail fold capillaroscopy examination was done in the office and was positive for active loops. A more macroscopic exam of the hands and fingers revealed hypervascularity of the cuticles bilaterally and irregular cracking along the lateral portions of the fingers (Figure 4; Figure 5). Labs that were completed prior to his visit to our clinic are shown in Table 1. Inspection and range of motion of all extremities did not reveal any abnormalities. Lung auscultation was completed and was clear to auscultation bilaterally. The patient was diagnosed with ADM. A chest X-ray was recommended to evaluate for ILD and was found to be normal. He was offered a high-resolution CT lung exam; however, he refused this. He was initially treated with hydroxychloroquine after obtaining clearance from an optometrist. The initial dose started at 200 mg, then increased to 300 mg based on 5 mg/kg body weight. This course of treatment was discontinued after four months as the patient did not have any improvement in his clinical course. He is currently on methotrexate 15 mg per week with supplemental daily folic acid at 1 mg per day and reports improvement in his joint pains, decreased rash, and increased general well-being. He is currently being followed in the rheumatology clinic for long-term progress.

**Table 1:** Patient’s laboratory test results

Laboratory Test	Patient Results	Normal Values
Antinuclear antibody	1:1280, speckled pattern	<1:40
C-reactive protein	<1 mg/L	0-10 mg/L
Rheumatoid factor	<10.0 IU/mL	<14.0 IU/mL
Sedimentation rate	10 mm/hr	0-30 mm/hr
HLA-B27	Negative	-
Lyme disease serology	Negative	-
Mitochondrial (M2) antibody	<20.0 units	0-20.0 units
Anti-EJ antibody	Negative	-
Anti-Jo-1 antibody	<20.0 units	<20.0 units
Anti-Ku antibody	Negative	-
Anti-MDA-5 antibody (CADM-140)	<20.0 units	<20.0 units
Anti-Mi-2 antibody	Negative	-
Anti-NXP-1 (P140) antibody	<20.0 units	<20.0 units
Anti-OJ antibody	Negative	-
Anti-PL-12 antibody	Negative	-
Anti-PL-7 antibody	Negative	-
Anti-PM/Scl-100 antibody	<20.0 units	<20.0 units
Anti-SAE1 antibody	<20.0 units	<20.0 units
Anti-SRP antibody	Negative	-
Anti-SS-A 52kD antibody	<20.0 units	<20.0 units
Anti-TIF-1-gamma antibody	<20.0 units	<20.0 units
Anti-U1 RNP antibody	<20.0 units	<20.0 units
Anti-U2 RNP antibody	Negative	-
Anti-U3 RNP (fibrillarin) antibody	Negative	-
Anti-CCP antibody	<2.0 units	0-19.0 units

## Discussion

The diagnosis of CADM is made clinically by the presence of a classic DM rash and relevant skin findings. The pathognomonic cutaneous characteristics of DM are Gottron’s papules, scaly plaques that appear over the knuckles of the hands, fingers, or elbows, and Gottron’s sign, a red flat rash over the fingers, elbows, and knees. Additional findings may include heliotrope rash, the shawl sign, V-sign, inflammatory arthritis of the distal interphalangeal joints, nail fold capillary abnormalities such as cuticle hypervascularity or telangiectasias, and mechanic’s hands. However, the complete absence of observable muscle involvement makes it challenging to distinguish the rash of CADM from other skin conditions.

Many skin disorders, such as psoriasis, eczema, and cutaneous lupus erythematosus (CLE), can have similar physical exam features. The use of skin biopsies can help to aid in the differentiation among the group. CADM skin biopsy findings include vacuolar changes of the basal layer, increased lymphocytic infiltrate, and increased mucin deposition in the dermis [7]. This differs from psoriasis, where key histological findings include hyperkeratosis, parakeratosis, loss of the granular cell layer, epidermal acanthosis, foci of neutrophils in the parakeratotic stratum corneum, and spongiform neutrophilic micropustules in the spinous layer of the epidermis [8]. Histological findings of eczema can be more nonspecific and can be based on the length of time a patient has been dealing with the condition [9]. The acute-stage eczema biopsy would show mild epidermal hyperplasia, infiltrations of lymphocytes and macrophages along the venous plexus of the dermis, and intercellular edema of the epidermis [9]. The chronic-stage eczema biopsy would show hyperplasia, hyperkeratosis, inflammatory cell infiltration with lymphocytes and macrophages, and a lack of intercellular edema [9]. The histological findings of CLE also differ based on the acuity of the disorder. Acute cutaneous lupus erythematosus (ACLE) histology shows sparse dermal cellular infiltrate, focal liquefactive degeneration of the basal epidermis, and upper dermal edema [10]. Subacute cutaneous lupus erythematosus (SCLE) demonstrates focal liquefactive degeneration, sparse mononuclear infiltrates in the upper one-third of the dermis, dermal edema, and infrequent epidermal necrosis [10]. ACLE and SCLE can both demonstrate varying degrees of hyperkeratosis, degeneration of the basal cell layer, and a mononuclear cell infiltrate at the dermal-epidermal junction and within the dermis [10].

CADM is relatively rare, and its presentation is highly variable. Some patients display many characteristic signs, while others show very few, with varying degrees of severity. For these reasons, the clinical diagnosis can be difficult and is often delayed or misdiagnosed. In our case, a diagnosis of CADM with a subset of IIM was made using the widely endorsed European League Against Rheumatism/American College of Rheumatology (EULAR/ACR) classification criteria. With the presence of both a heliotrope rash and Gottron’s papules, a score of 7.3 was obtained, establishing the diagnosis with nearly 90% positive predictive value [1].

Once a diagnosis is established, common treatment options include steroids, disease-modifying antirheumatic drugs (DMARDs), or other immunosuppressive therapies. Some experts advise against using immunosuppressive drugs or DMARDs if there is no muscle involvement [11]. In contrast, hydroxychloroquine as a monotherapy has shown significant improvement in approximately 70% of CADM patients in one study, as measured by the Cutaneous Dermatomyositis Disease Area and Severity Index (CDASI) [3]. A combination of steroids, antimalarials, and immunosuppressants has been used with varying efficacy. Patients can be started on multiple types of treatments as mentioned above. Similar to our patient, hydroxychloroquine can be a starting medication. However, if medications like this do not provide relief of symptoms, patients can be transitioned to one or more of the options that were mentioned above.

The prognosis of CADM is often chronic and incurable, even with treatment. After accounting for a 10% mortality rate, only 20% achieved remission, and 80% experienced chronic or recurrent manifestations [7]. Several factors are associated with a worse outcome, the most significant being the presence of an underlying malignancy or ILD. The malignancies that are commonly associated with DM include lung, ovarian, breast, colorectal, cervical, bladder, nasopharyngeal, esophageal, pancreatic, kidney, and hematologic [12,13]. Furthermore, clinicians should be aware that a small minority of patients may eventually develop late-onset muscle weakness. Interestingly, several studies suggest an increased frequency of internal malignancy and ILD associated with CADM compared to classic DM. However, the extent of this relative risk is unclear, and approximately one-third of patients do develop ILD [7].

## Conclusions

CADM is an uncommon disease that can present in a myriad of ways. This case of CADM is presented to raise awareness among clinicians so that pathognomonic cutaneous characteristics of DM, such as Gottron’s papules and Gottron’s sign, are not overlooked. By recognizing these key features, we aim to help clinicians make a more timely diagnosis, reduce the delay in necessary screening tests, initiate a treatment protocol more quickly, and provide insight into the potential underlying malignant causes of DM. In doing so, we hope clinicians can improve the patient’s clinical course and minimize complications arising from the disease.
